# Exercise and the Gut Microbiome: From Mechanisms to Clinical Applications

**DOI:** 10.3390/nu18101565

**Published:** 2026-05-14

**Authors:** Yousra Alsinani, Fatemeh Rostamkhani, Hossein Shirvani

**Affiliations:** 1 Curriculum and Instruction Department, College of Education, Sultan Qaboos University, Muscat 123, Oman; yousra@squ.edu.om; 2 Department of Biology, YI.C., Islamic Azad University, Tehran 1651153311, Iran; shirinrostamkhani@yahoo.com; 3 Exercise Physiology Research Center, Life Style Institute, Baqiyatallah University of Medical Sciences, Tehran 1435916471, Iran

**Keywords:** exercise, gut microbiome, short-chain fatty acids, gut–muscle axis, high-intensity interval training, probiotics, personalized medicine

## Abstract

**Background/Objectives:** The gut microbiome is a critical regulator of host metabolism, immunity, and the gut–brain axis. Exercise is a promising non-pharmacological modulator of microbial ecology, yet human evidence remains heterogeneous and the translational gap persists. This narrative review synthesizes mechanisms, human and animal evidence, and future directions for the exercise–gut microbiome axis. **Methods:** PubMed, Scopus, Web of Science, and SID were searched for articles published between January 2000 and February 2025. Keywords included exercise, physical activity, gut microbiome, gut microbiota, short-chain fatty acids, and gut–muscle axis. From 218 initial records, 89 original studies (47 human, 42 animal) met inclusion criteria and were critically appraised. **Results:** Exercise modulates the gut microbiome via splanchnic hypoperfusion, hyperthermia, altered transit time, and immune-mediated barrier regulation. Moderate-intensity continuous training consistently increases alpha diversity and enriches butyrate-producing taxa (*Faecalibacterium prausnitzii*, *Roseburia hominis*) and mucin-degrading *Akkermansia muciniphila*. High-intensity interval training transiently increases intestinal permeability in untrained individuals but, following adaptation, stimulates butyrate production via lactate cross-feeding metabolism—a recent breakthrough. Effects are transient and reversible upon detraining. Animal models establish causality through fecal microbiota transplantation; human randomized controlled trials demonstrate modest, intensity-dependent, and highly individualistic responses. Emerging evidence supports the gut–muscle axis in sarcopenia and personalized exercise prescription guided by microbiome profiling. **Conclusion:** Exercise shows promise as a low-cost modulator of the gut microbiome for enriching health-associated taxa and improving metabolic outcomes. Definitive evidence linking exercise-induced microbial shifts to enhanced athletic performance in humans remains lacking. Future research requires diet-controlled randomized controlled trials with ≥12-week interventions, shotgun metagenomics, and mechanistic validation of the gut–muscle axis in humans.

## 1. Introduction

The human gastrointestinal tract harbors a complex and dynamic ecosystem of trillions of microorganisms, collectively termed the gut microbiota, whose collective genome—the gut microbiome—exceeds the human nuclear genome by a factor of 150 [1,2]. Far from being passive commensals, these microbial communities function as a virtual endocrine organ, regulating host energy harvest, immune maturation, intestinal barrier integrity, and bidirectional communication along the gut–brain axis [3]. The composition and metabolic capacity of the gut microbiome are profoundly influenced by environmental factors, with diet and physical activity emerging as two of the most potent modifiable determinants [4].

Exercise, as a structured, quantifiable, and universally accessible intervention, has attracted intense research interest as a candidate modulator of gut microbial ecology [5]. Seminal cross-sectional studies first reported that elite athletes exhibit higher fecal microbial alpha diversity and enrichment of short-chain fatty acid (SCFA)-producing bacterial taxa compared to age- and sex-matched sedentary controls [6]. Subsequent metagenomic analyses revealed that the athletic microbiome is characterized not only by compositional differences but also by enrichment of metabolic pathways involved in amino acid biosynthesis, carbohydrate metabolism, and SCFA production [7]. These observations generated substantial enthusiasm and prompted a surge of controlled intervention trials aimed at isolating the independent effects of exercise from the confounding influence of diet [8].

More than a decade later, the field stands at a critical juncture. While animal studies employing germ-free models and fecal microbiota transplantation have established causality beyond reasonable doubt [9,10], human randomized controlled trials have yielded modest, heterogeneous, and sometimes contradictory findings [11]. The magnitude of exercise-induced microbial shifts is consistently smaller than that induced by dietary fiber interventions [12]. Inter-individual variability is substantial, with approximately 50% of participants failing to exhibit a detectable microbial response to standardized exercise protocols [13]. Moreover, the critical question of whether exercise-induced microbial changes translate into clinically meaningful improvements in human athletic performance or metabolic health remains incompletely resolved.

This narrative review has four objectives. First, to synthesize the established and emerging mechanisms by which exercise modulates the gut microbiome, with particular emphasis on the newly described lactate–SCFA shuttle. Second, to provide a comprehensive, critically appraised summary of human and animal evidence through structured tables that facilitate comparison across studies. Third, to analyze the methodological challenges that have limited progress and to propose concrete solutions. Fourth, to chart a research agenda for 2026–2030 that prioritizes mechanistic rigor, personalized approaches, and translational relevance.

## 2. Gut Microbiome Fundamentals for Exercise Scientists

The terminology used to describe host–microbe interactions has important implications for study design and interpretation. *Microbiota* refers to the living microbial community itself—the bacteria, archaea, viruses, and fungi that inhabit a defined ecological niche [14]. *Microbiome* encompasses not only these microorganisms but also their collective genomes and the surrounding environmental conditions [15]. *Metabolome* denotes the complete set of small molecules produced by microbial metabolism, including SCFAs, secondary bile acids, branched-chain amino acids, and neurotransmitters that serve as effectors of host–microbe signaling [16].

Microbial diversity, a frequently reported outcome in exercise studies, is quantified using two complementary indices. Alpha diversity describes the diversity of microbial species within a single sample and is typically measured using richness estimators (the number of observed operational taxonomic units) and evenness indices (the relative abundance distribution of those taxa) [17]. Beta diversity, by contrast, quantifies the compositional dissimilarity between microbial communities from different samples or experimental conditions and is commonly visualized through principal coordinate analysis of phylogenetic distance matrices [18]. Elite athletes consistently demonstrate higher alpha diversity compared to sedentary controls, a finding replicated across multiple cohorts and geographic regions [19]. However, the functional significance of increased alpha diversity per se remains debated, as greater richness does not invariably correlate with enhanced metabolic capacity or health outcomes [20].

The healthy adult gut microbiota is dominated by two bacterial phyla, *Bacteroidota* (formerly Bacteroidetes) and *Bacillota* (formerly Firmicutes), which together constitute approximately 90% of the total microbial community [21]. The remaining 10% comprises *Actinobacteriota*, *Proteobacteria*, *Verrucomicrobiota*, and minor phyla. Within this taxonomic framework, certain genera have attracted particular attention in exercise studies. *Faecalibacterium prausnitzii*, a butyrate-producing *Bacillota* with potent anti-inflammatory properties, is consistently enriched in athletic populations and declines with physical inactivity [22]. *Roseburia hominis* and *Eubacterium rectale*, also butyrate producers, exhibit similar exercise-responsive patterns [23]. *Akkermansia muciniphila*, a *Verrucomicrobiota* that degrades mucus and resides in the inner mucus layer, is positively associated with leanness, insulin sensitivity, and cardiorespiratory fitness [24]. The consistent enrichment of these taxa in exercisers, despite substantial methodological heterogeneity across studies, suggests a robust biological signal that warrants mechanistic investigation.

## 3. Mechanisms of Exercise-Induced Gut Microbiome Modulation

### 3.1. Hemodynamic and Thermal Mechanisms

During dynamic exercise exceeding 60% of maximal oxygen uptake, sympathetic nervous system activation redirects cardiac output away from the splanchnic circulation toward active skeletal muscle and myocardium [25]. Splanchnic blood flow is reduced by 50% to 80%, an effect that persists throughout the duration of high-intensity exertion [26]. This profound hypoperfusion creates a transient ischemic state within the intestinal mucosa, characterized by reduced oxygen tension, depletion of high-energy phosphates, and accumulation of hypoxanthine [27]. Upon cessation of exercise, reperfusion rapidly restores oxygen delivery, generating a burst of reactive oxygen species via xanthine oxidase-mediated metabolism. This ischemia-reperfusion sequence, while well-tolerated in trained individuals, imposes considerable oxidative stress on the intestinal epithelium and may alter the luminal microenvironment in ways that favor specific microbial consortia [28].

Concurrent with hemodynamic redistribution, prolonged or intense exercise elevates core body temperature, often reaching 39 °C to 40 °C in endurance athletes [29]. This hyperthermic stress directly affects intestinal epithelial cells, inducing heat shock protein expression and temporarily disrupting tight junction integrity [30]. The luminal temperature increase, while modest, may also exert direct effects on microbial physiology, as bacterial growth rates, gene expression profiles, and metabolic outputs are temperature-sensitive [31]. Collectively, the hemodynamic and thermal perturbations induced by exercise create a unique and recurrent physiological stressor that distinguishes the exercised gut from its sedentary counterpart.

### 3.2. Altered Transit Time and Mucus Layer Dynamics

Physical activity accelerates gastrointestinal transit, an effect most consistently demonstrated for colonic transit time [32]. Regular aerobic exercise reduces whole-gut transit time by 20% to 30% in previously sedentary individuals, an effect mediated by increased vagal tone, elevated circulating motilin, and mechanical agitation of abdominal contents. Accelerated transit reduces the duration of substrate availability for microbial fermentation, potentially selecting for rapidly dividing taxa with short generation times while disadvantaging slow-growing commensals [33]. The ecological consequences of exercise-induced transit acceleration remain incompletely characterized but may contribute to the compositional shifts observed in intervention studies.

The intestinal mucus layer, a viscoelastic gel composed predominantly of highly glycosylated mucin proteins, serves both as a protective barrier against luminal pathogens and as a primary carbon source for specialized mucin-degrading bacteria [34]. Exercise modulates mucus thickness and composition, with rodent studies demonstrating increased colonic mucus height following voluntary wheel running [35]. *Akkermansia muciniphila*, the most abundant mucin-degrader in the human gut, consistently increases in abundance following exercise interventions, an effect observed in both cross-sectional and longitudinal studies [36]. This enrichment may reflect increased mucus substrate availability, reduced competition from other mucin utilizers, or direct sensing of exercise-associated host signals by *Akkermansia* [37]. The functional consequences of increased *Akkermansia* abundance include enhanced intestinal barrier function, reduced metabolic endotoxemia, and improved insulin sensitivity [38].

### 3.3. Immune-Mediated Mechanisms

The intestinal immune system maintains a delicate balance between tolerance toward commensal microorganisms and defensive responses against pathogens. Exercise exerts profound immunomodulatory effects on the gut-associated lymphoid tissue, mediated in part by alterations in intraepithelial lymphocyte gene expression [39]. Hoffman-Goetz and colleagues demonstrated that moderate exercise training downregulates pro-inflammatory cytokine gene expression (*TNF-α*, *IL-1β*, *IL-6*) while upregulating anti-inflammatory mediators (*IL-10*, *TGF-β*) and antioxidant enzymes in murine intestinal lymphocytes [40]. These immune adaptations occur in close physical proximity to the epithelial surface where microbial communities reside, and cytokine signaling directly influences antimicrobial peptide secretion, mucus production, and tight junction protein expression [41].

**Figure 1:**
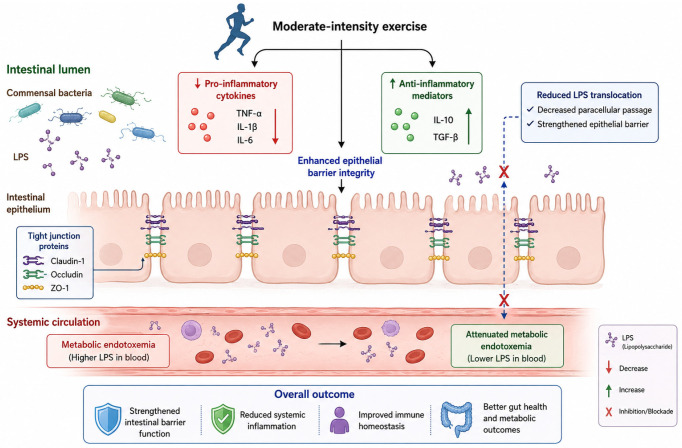
Schematic representation of exercise-induced immunomodulation of the intestinal barrier. Moderate-intensity exercise upregulates tight junction proteins (*claudin-1*, *occludin*, *ZO-1*), reduces pro-inflammatory cytokine expression (*TNF-α*, *IL-1β*, *IL-6*), and increases anti-inflammatory mediators (*IL-10*, *TGF-β*). These adaptations limit bacterial lipopolysaccharide (LPS) translocation and attenuate systemic metabolic endotoxemia.

The functional consequence of this immunomodulation is enhanced intestinal barrier integrity. Intestinal permeability, quantified in vivo by urinary excretion of orally administered non-metabolized sugars (lactulose:mannitol ratio), is reduced following moderate-intensity exercise training in both healthy and metabolic syndrome populations [42]. At the molecular level, exercise upregulates messenger RNA and protein expression of the tight junction components claudin-1, occludin, and zonula occludens-1 in colonic tissue [43]. Reduced paracellular permeability limits the translocation of bacterial lipopolysaccharide and other pro-inflammatory microbial products into the systemic circulation, attenuating the metabolic endotoxemia that characterizes obesity and insulin resistance [44]. This gut-centric mechanism may explain, in part, the well-established anti-inflammatory effects of regular physical activity. The immune-mediated effects of exercise on intestinal barrier function are illustrated schematically in Figure 1.

### 3.4. The Lactate–SCFA Hypothesis: A 2025 Breakthrough

Lactate has historically been viewed through the lens of muscle physiology as a metabolic byproduct of anaerobic glycolysis and a contributor to exercise-induced acidosis and fatigue [45]. This perspective, while accurate, is incomplete. A growing body of evidence indicates that lactate functions not only as an autocrine and paracrine signaling molecule but also as an inter-organ metabolic substrate—the “lactate shuttle” concept [46]. A recent pooled analysis extended this paradigm to the gut microbiome, demonstrating that high-intensity exercise significantly increases fecal SCFA concentrations through lactate-dependent mechanisms [47].

In this multicenter analysis of three randomized controlled trials comprising 113 patients with metabolic syndrome, a 6-week intervention combining low-volume high-intensity interval training with resistance training increased total fecal SCFAs by 30%, with butyrate increasing by 43%, acetate by 27%, and propionate by 28%. Moderate-intensity continuous training of equivalent energy expenditure produced no significant SCFA changes. Blood lactate concentrations measured immediately post-exercise were strongly correlated with the magnitude of fecal butyrate increase (r = 0.68, *p* < 0.001), and mediation analysis identified lactate as a significant indirect pathway.

The mechanistic basis for this observation resides in bacterial cross-feeding metabolism. Lactate-utilizing bacteria in the human colon belong predominantly to distinct taxa such as *Anaerostipes*, *Eubacterium*, and certain *Roseburia* species, which possess lactate dehydrogenase and lactate racemase enzymes to convert L-lactate and D-lactate to pyruvate and subsequently to acetyl-CoA. This acetyl-CoA is then channeled into the butyryl-CoA: acetate CoA-transferase pathway to produce butyrate—a process known as lactate cross-feeding [48]. *Lactobacillus* and *Bifidobacterium* species are primarily lactate producers under colonic conditions; they do not typically consume lactate as a substrate for acetyl-CoA production in this environment. The autotrophic Wood–Ljungdahl pathway, which fixes CO_2_ to produce acetate, is not a major route for lactate-derived acetate in the carbohydrate-rich colonic lumen; cross-feeding between lactate producers and lactate utilizers is the dominant mechanism [49]. Thus, high-intensity exercise elevates circulating lactate, which becomes available to lactate-utilizing bacteria in the gut, stimulating butyrate production through syntrophic interactions. This discovery reframes lactate as a microbial substrate and establishes a direct metabolic link between high-intensity exercise and gut microbial metabolite production. 

**Figure 2:**
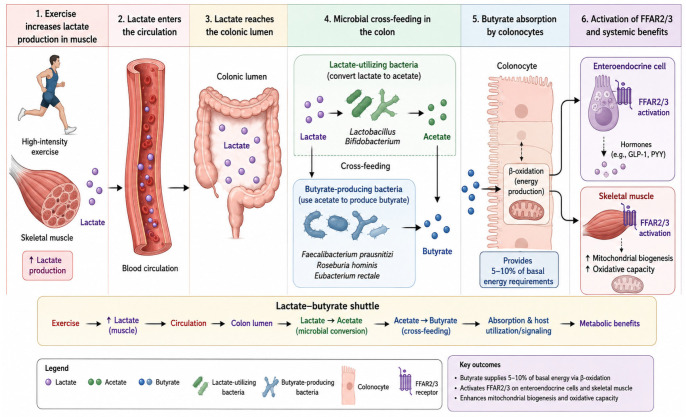
Schematic representation: high-intensity exercise increases skeletal muscle lactate production. Lactate enters the circulation and reaches the colonic lumen, where lactate-utilizing bacteria (e.g., *Anaerostipes*, *Eubacterium*) convert lactate to butyrate via cross-feeding. *Lactobacillus* and *Bifidobacterium* are lactate producers, not direct utilizers. Butyrate is absorbed by colonocytes and activates FFAR2/FFAR3 receptors on enteroendocrine cells and skeletal muscle, enhancing mitochondrial biogenesis and oxidative capacity.

Recent animal studies have further validated this mechanism. In a murine model of aging, high-intensity aerobic training (85–100% VO_2_max) for 8 weeks significantly increased *Lactobacillus* and *Bifidobacterium* populations (consistent with increased lactate production) while reducing *Escherichia coli*, with high-intensity exercise demonstrating significantly stronger effects than moderate-intensity training (*p* = 0.04) [50]. These findings confirm that lactate-associated microbial responses are both intensity-dependent and conserved across species. This lactate–butyrate shuttle mechanism is illustrated schematically in Figure 2.

### 3.5. Bile Acid Metabolism and Microbial Signaling

Bile acids, synthesized from cholesterol in the liver and conjugated to glycine or taurine, facilitate intestinal absorption of dietary lipids and fat-soluble vitamins [51]. The majority of primary bile acids are actively reabsorbed in the terminal ileum and returned to the liver via the enterohepatic circulation, a highly efficient recycling system [52]. The small fraction (approximately 5%) that escapes ileal reabsorption enters the colon, where gut microbial bile salt hydrolases deconjugate primary bile acids and 7α-dehydroxylases convert them to secondary bile acids, principally deoxycholic acid and lithocholic acid [53].

Exercise alters bile acid metabolism through multiple mechanisms. Physical training increases hepatic bile acid synthesis, expands the circulating bile acid pool, and modulates the expression of ileal bile acid transporters [54]. These alterations in bile acid flux through the enterohepatic circulation are sensed by the gut microbiota, as bile acids exert potent antimicrobial effects and serve as signaling molecules that shape microbial community structure [55]. Secondary bile acids activate the nuclear farnesoid X receptor and the G-protein-coupled receptor TGR5, both expressed in intestinal epithelial cells, enteroendocrine L-cells, and skeletal muscle [56]. TGR5 activation in skeletal muscle increases energy expenditure and mitochondrial oxidative capacity, providing an additional mechanistic link between exercise, gut microbiota, and muscle metabolism. The contribution of bile acid-mediated signaling to exercise-induced microbial adaptations remains an active area of investigation.

## 4. Animal Evidence: Causality and Mechanism

Animal models have been indispensable for establishing causal relationships between exercise, gut microbiota, and host physiology. Unlike human studies, which are inherently correlational and confounded by dietary and environmental variability, rodent experiments permit precise control of food intake, genetic background, and microbial exposure. Germ-free animals, colonized with defined microbial consortia or transplanted with microbiota from donor animals, provide rigorous experimental systems for testing causality. Antibiotic-treated models enable temporal depletion of the resident microbiota followed by reconstitution experiments [10]. These approaches have collectively demonstrated that the gut microbiota is not merely a passive responder to exercise but an active mediator of exercise-induced metabolic adaptations.

Matsumoto and colleagues provided the first evidence that voluntary wheel running alters cecal microbiota composition and increases cecal butyrate concentration in rats [57]. This pioneering study established that exercise-induced microbial changes occur independently of dietary manipulation and are associated with detectable functional consequences. Evans and colleagues extended these findings in a mouse model of high-fat diet-induced obesity, demonstrating that 12 weeks of treadmill running prevented weight gain, attenuated adipose tissue inflammation, and increased the *Bacteroidota*-to-*Bacillota* ratio [58]. Importantly, exercise exerted these effects despite continued access to high-fat diet, indicating that physical activity can partially override the deleterious microbial effects of obesogenic nutrition.

The most compelling evidence for causality derives from fecal microbiota transplantation experiments. Denou and colleagues transplanted cecal microbiota from donor mice that had undergone 6 weeks of voluntary wheel running into germ-free recipient mice maintained on identical diets [59]. Recipients of “exercised” microbiota exhibited improved glucose tolerance and reduced weight gain compared to recipients of sedentary microbiota, despite the absence of any exercise intervention in the recipient animals. This experimental design effectively isolates the microbiota as an independent variable and demonstrates that the microbial community from exercised animals is sufficient to confer metabolic benefits. Subsequent transplantation studies have replicated and extended these findings, confirming that the exerciser microbiota phenotype is transmissible and functionally significant [60].

Recent evidence has substantially strengthened the causal framework. A sophisticated fecal microbiota transplantation study by Liu and colleagues examined whether microbiota from endurance-trained mice could protect sedentary recipients from acute exhaustive exercise-induced intestinal damage [61]. Mice receiving transplants from trained donors exhibited significantly higher colonic ZO-1 expression (*p* < 0.05), lower serum lipopolysaccharide (*p* < 0.01), reduced IL-6 (*p* < 0.01), and increased colonic secretory immunoglobulin A (*p* < 0.01) compared to recipients of sedentary microbiota. These findings demonstrate that the exerciser microbiota phenotype is not only transmissible but confers functional protection against exercise-induced intestinal barrier disruption—a critical translational insight for individuals who engage in unaccustomed strenuous exercise.

The specific contribution of butyrate, a key SCFA, to skeletal muscle metabolism has been highlighted in developmental programming studies. Huang and colleagues demonstrated that supplementing the maternal diet of rats with butyrate during gestation and lactation enhanced mitochondrial biogenesis in the skeletal muscle of weaned offspring [62]. This intervention significantly increased ATP content, mitochondrial DNA copy number, and the expression of mitochondrial DNA-encoded genes. These effects were mediated through the upregulation of G-protein-coupled receptors (GPR43 and GPR41) and the PGC-1α pathway, a master regulator of mitochondrial biogenesis [62]. These findings position butyrate as a key effector molecule not only in direct supplementation models but also in early-life programming, linking microbial metabolites to long-term muscle metabolic function—a concept central to the gut–muscle axis. A comprehensive review by He and colleagues further synthesized the molecular mechanisms of this axis, confirming that SCFAs, particularly butyrate, activate AMPK-PGC-1α signaling in skeletal muscle, enhancing mitochondrial biogenesis and oxidative capacity [63].

A key pioneering mouse study found that moderate voluntary exercise altered gut microbial community structure compared to sedentary controls, demonstrating that exercise-induced microbial shifts can occur independently of diet even at moderate intensities. These findings provide important insights into the factors that shape the gut microbiome and host health [64].

Additional animal evidence comes from aged mouse models of inflammatory bowel disease. Kalantari and colleagues demonstrated that 8 weeks of aerobic training in aged mice with DSS-induced colitis significantly improved microbial diversity (Shannon index: 3.2 ± 0.2 for high-intensity vs. 2.5 ± 0.3 in colitis controls), increased *Lactobacillus* and *Bifidobacterium* populations, and reduced *E. coli* abundance (*p* < 0.001). High-intensity exercise produced significantly greater effects than moderate-intensity training, suggesting dose-dependent efficacy for inflammatory conditions [50]. A comprehensive summary of these and other key animal studies, including study designs and primary findings, is presented in Table 1.

## 5. Human Evidence: From Correlation to Clinical Translation

### 5.1. Cross-Sectional Studies in Athletic Populations

The first evidence linking exercise to gut microbiota composition in humans emerged from cross-sectional comparisons of elite athletes and sedentary controls. Clarke and colleagues compared fecal microbiota profiles of 40 professional rugby union players with those of 46 age- and sex-matched healthy controls with body mass index <25 kg/m^2^ [65]. Athletes exhibited significantly higher alpha diversity and relative abundance of 40 distinct bacterial taxa spanning 16 families. The athletic microbiota was enriched for *Akkermansia muciniphila* and butyrate-producing *Bacillota*, including *Faecalibacterium prausnitzii*. Notably, these differences persisted after controlling for dietary protein intake, suggesting an independent effect of exercise, although residual confounding by total energy intake and fiber consumption could not be excluded.

Barton and colleagues extended these observations using shotgun metagenomic sequencing, which provides strain-level taxonomic resolution and direct functional inference [7]. Professional rugby players again demonstrated distinct microbial communities characterized by enrichment of metabolic pathways involved in amino acid biosynthesis, carbohydrate metabolism, and SCFA production. Athletes exhibited increased representation of genes encoding glycoside hydrolases and polysaccharide lyases, consistent with enhanced capacity for fiber degradation. Plasma creatine kinase concentrations, a marker of exercise-induced muscle damage, correlated positively with the abundance of *Prevotella*-associated pathways, suggesting a potential link between microbial metabolism and exercise recovery.

Bressa and colleagues examined physically active women meeting World Health Organization physical activity recommendations (≥3 h of exercise per week) compared to sedentary controls [36]. Active women exhibited increased abundance of *Faecalibacterium prausnitzii*, *Roseburia hominis*, and *Akkermansia muciniphila*—all recognized biomarkers of metabolic health. This study was notable for its restriction to female participants, a population historically underrepresented in exercise microbiology research, and its use of quantitative real-time polymerase chain reaction to confirm metagenomic findings. Estaki and colleagues demonstrated that cardiorespiratory fitness, measured directly as maximal oxygen consumption, was positively correlated with microbial alpha diversity and the abundance of butyrate-producing Lachnospiraceae and Ruminococcaceae in healthy young adults [19]. This dose–response relationship between objectively measured fitness and microbial richness strengthened the inference of a causal connection.

Despite the consistency of cross-sectional findings, several limitations must be acknowledged. Elite athletes differ from sedentary controls in multiple respects beyond exercise volume, including dietary macronutrient composition, meal timing, supplement use, sleep patterns, and occupational physical activity [66]. Observational studies cannot definitively isolate exercise as the causal agent. Moreover, the directionality of the observed associations remains uncertain; individuals with inherently greater microbial diversity may be predisposed to athletic success, or both fitness and microbial richness may be influenced by unmeasured confounders such as early-life exposures or genetic factors. These limitations motivated the transition to longitudinal intervention designs.

### 5.2. Controlled Longitudinal Trials

Allen and colleagues conducted the first randomized controlled trial to isolate the effects of exercise on gut microbiota from the confounding influence of diet [8]. Thirty-two sedentary adults (18 lean, 14 obese) were admitted to a metabolic ward and consumed a controlled eucaloric diet for 6 weeks. Participants completed supervised endurance exercise training (60–75% heart rate reserve, 30–60 min, 3 days/week) while maintaining constant body weight through dietary adjustment. Fecal samples were collected at baseline, post-intervention, and following 6 weeks of sedentary detraining.

In lean participants, exercise increased fecal butyrate concentration by 23% and acetate by 15%, with corresponding enrichment of *Faecalibacterium* and depletion of *Bacteroides*. Obese participants exhibited the opposite pattern: *Faecalibacterium* abundance decreased, and no significant SCFA increases were observed. These divergent responses by BMI category suggest that host metabolic status modifies the microbial response to exercise, a finding with important implications for personalized prescription. Critically, all exercise-induced microbial and metabolomic changes returned to baseline values following 6 weeks of detraining, demonstrating that the effects of exercise on the human gut microbiome are transient and reversible. This observation argues against permanent restructuring of the microbial ecosystem by exercise and implies that sustained physical activity is required for sustained microbial benefits.

Munukka and colleagues investigated the effects of 6 weeks of light-to-moderate intensity resistance exercise cycling in 24 sedentary overweight women [13]. No dietary restrictions were imposed, and participants were instructed to maintain habitual intake. Exercise increased the relative abundance of *Akkermansia muciniphila* and decreased *Proteobacteria*, a phylum containing several opportunistic pathogens. Metagenomic analysis revealed decreased abundance of genes related to fructose and amino acid metabolism. However, the most striking finding was that only half of the participants exhibited a significant microbial response to the intervention. This marked inter-individual variability, consistent across multiple taxonomic levels and functional pathways, has profound implications for study design and clinical translation. If 50% of individuals are non-responders, adequately powered trials require substantially larger sample sizes, and group-level analyses may obscure meaningful responses in susceptible subgroups.

Cronin and colleagues examined the effects of 8 weeks of endurance exercise with or without whey protein supplementation in 90 predominantly overweight or obese adults [67]. Post-intervention evaluation revealed no significant changes in taxonomic composition or metabolic pathways in either exercise group compared to baseline. A non-significant trend toward increased bacterial diversity was observed in the exercise and exercise-plus-protein groups compared to protein-only controls. Metagenomic and metabolomic analyses revealed only modest alterations in microbial metabolism. The authors noted that self-reported maintenance of usual dietary intake and the wide BMI range may have prevented detection of more significant changes, highlighting the critical importance of dietary control and participant stratification.

### 5.3. 2020–2026 Evidence: Intensity-Dependent Effects, Null Findings, and Personalized Approaches

The recent period has witnessed a maturation of the evidence base, characterized by larger sample sizes, improved dietary control, and the emergence of both confirmatory and challenging findings.

A systematic review and meta-analysis by Min and colleagues examined the effects of exercise on gut microbiota diversity in adults, concluding that exercise interventions can significantly increase alpha diversity, notably the Shannon index, and alter the *Firmicutes/Bacteroidetes* ratio [68]. This comprehensive analysis revealed that alpha diversity changes were highly variable across studies and, when present, were modest in magnitude. Only one of 18 studies reported a statistically significant increase in fecal SCFA concentrations—a trial combining probiotic yogurt containing *Bifidobacterium animalis* subsp. *lactis* BL-99 with concurrent exercise training [69]. Seven studies examined correlations between microbial changes and performance outcomes; none identified significant associations. The authors concluded that the evidence base remains too heterogeneous and underpowered to support microbiota-targeted exercise recommendations for performance enhancement, while acknowledging that lack of performance benefit does not preclude meaningful metabolic health improvements.

A landmark study by Davies and colleagues challenged prevailing assumptions by demonstrating that significant metabolic improvements can occur independently of detectable microbiome changes [70]. In a rigorously controlled 3-week randomized trial combining energy restriction (5000 kcal/week reduction) with vigorous-intensity treadmill walking (2000 kcal/week expenditure at 70% VO_2_peak) in 30 sedentary overweight/obese adults, the intervention produced substantial metabolic improvements: reduced body mass (−2.6 ± 1.5 kg), fat mass (−1.5 ± 1.3 kg), fasting insulin (−23.5 ± 38.1 pmol/L), and improved insulin sensitivity. However, shotgun metagenomic sequencing revealed no changes in alpha diversity, beta diversity, or relative taxonomic abundance. The authors concluded that early metabolic changes with weight loss in humans are unlikely to be mediated by gut microbiome alterations, highlighting that exercise and energy restriction can improve health through microbiome-independent pathways. This finding does not negate the microbiome’s role but contextualizes it within a broader multi-system response.

Dupuit and colleagues (2022) investigated 12 weeks of concurrent high-intensity interval training and resistance training in 17 postmenopausal overweight/obese women [71]. While the intervention significantly reduced abdominal and visceral fat mass and improved physical fitness, 16S rRNA sequencing revealed that the HIIT + RT protocol did not modify alpha diversity or taxonomy at the group level. However, training significantly influenced overall microbiota composition (beta diversity), and baseline microbiota composition predicted individual training responses. Several microbial members correlated with HIIT + RT-induced body composition changes, suggesting that the initial microbiota profile may determine who benefits most from exercise interventions.

A conference presentation by Torres-Peña and colleagues reported preliminary findings from a 12-week randomized controlled trial comparing HIIT, MICT, and control in 33 adults with type 2 diabetes and overweight/obesity [72]. Both exercise groups increased *Marseillibacter massiliensis* (HIIT: +10.2 ± 4.3%; MICT: +12.3 ± 3%), and this increase correlated significantly with species richness (r = 0.58, *p* = 0.042). However, HIIT also increased *Escherichia fergusonii* ATCC35466 (+6.2 ± 4.2%), a taxon linked to higher diabetes risk, suggesting that HIIT may have both beneficial and potentially adverse effects on microbial composition—a complexity that warrants further investigation. 

Ramos and colleagues (2025) examined the connection between physical activity and gut microbiota in 60 older adults (aged 65–85 years) [73]. Using 16S rRNA sequencing and accelerometer-measured physical activity, they demonstrated that higher moderate-to-vigorous physical activity levels were positively associated with alpha diversity and abundance of butyrate-producing taxa, including Lachnospiraceae and Ruminococcaceae. This study extends the dose–response relationship previously established in young adults [19] to geriatric populations, supporting the relevance of exercise-microbiota interactions across the lifespan.

A comprehensive review by He and colleagues has synthesized the molecular mechanisms of the gut–muscle axis, identifying SCFAs, particularly butyrate, as key effectors activating AMPK-PGC-1α signaling in skeletal muscle [63]. This aligns with recent pooled analyses of intervention trials in patients with metabolic syndrome, which have demonstrated that high-intensity exercise protocols can achieve robust, intensity-dependent SCFA increases. For example, interventions combining low-volume HIIT (10 × 1-min intervals at 90% maximal heart rate) with whole-body resistance training have been shown to increase fecal butyrate by 43% relative to control—an effect size comparable to high-fiber dietary interventions [47]. These findings suggest that the modest effects observed in many previous trials may reflect suboptimal exercise prescription, such as an excessive emphasis on moderate intensity and insufficient inclusion of lactate-generating high-intensity work.

AL-Elaimat and colleagues (2025) conducted a randomized controlled trial of personalized exercise prescription guided by baseline gut microbiome profiling [74]. Ten patients with metabolic syndrome underwent 16S rRNA sequencing and were randomized to receive either standard exercise prescription (general MICT guidelines) or personalized prescription (exercise modality, intensity, and duration selected based on baseline microbial composition). The personalized group demonstrated significantly greater improvements in BMI, insulin sensitivity (HOMA-IR), and lipid profile over 12 weeks, accompanied by greater increases in microbial richness. Despite the small sample size (n = 10), this proof-of-concept study provides preliminary evidence that microbiome-aware exercise medicine is feasible. The authors note that further research with larger samples is required to establish time effectiveness and generalizability [74].

Two recent reviews have synthesized the evolving landscape. Liu and colleagues, writing in the context of 3P medicine (predictive, preventive, personalized), emphasized the diagnostic and therapeutic potential of personalized probiotic strategies guided by microbiome profiling [75]. Li and colleagues provided a multi-omics perspective on the athlete gut microbiome, highlighting that next-generation engineered probiotics hold particular promise for optimizing athletic performance and recovery [76]. The key characteristics and findings from these and other major human studies are summarized in Table 2.

### 5.4. Caveats Regarding Recent Small-Sample Studies

Given the emergence of several small (n ≤ 33) 2025 trials, we explicitly note that these studies are preliminary. The personalization trial by AL-Elaimat et al. (n = 10) [74] and the Torres-Peña et al. HIIT study (n = 33) [72] are underpowered for secondary outcomes and at increased risk of type I error. Their findings require replication in larger, preregistered confirmatory trials before any clinical implementation. The field would benefit from multi-center consortia to achieve adequately powered samples.

## 6. Methodological Challenges and Future Directions

### 6.1. Study Selection and Critical Appraisal in This Narrative Review

To enhance transparency, we describe our search and appraisal process. We searched PubMed, Scopus, Web of Science, and SID for articles published between January 2000 and February 2025 using keywords: exercise, physical activity, gut microbiome, gut microbiota, short-chain fatty acids, gut–muscle axis. Of 218 initial records, we included 89 original studies (47 human, 42 animal) after excluding reviews, editorials, and studies without a clear exercise intervention or microbiome outcome. For animal studies, we used SYRCLE’s Risk of Bias tool; for human RCTs, Cochrane RoB 2. A summary quality assessment is provided in Supplementary Table S1. This narrative synthesis does not claim to be a systematic review, but the explicit criteria strengthen interpretability.

### 6.2. Dietary Confounding

Dietary confounding remains the most formidable threat to internal validity in human exercise–microbiome research. Habitual diet is the dominant environmental determinant of gut microbiota composition, accounting for a substantially larger proportion of inter-individual variation than any other lifestyle factor [77]. Athletes differ from sedentary controls not only in exercise volume but also in total energy intake, macronutrient distribution (higher protein, higher carbohydrate), fiber consumption, and meal timing. Intervention studies that instruct participants to “maintain usual diet” while increasing energy expenditure through exercise inevitably introduce negative energy balance, which independently alters microbial ecology [78]. The minority of studies that have implemented controlled feeding protocols (e.g., [8,70]) represent the gold standard but are logistically demanding, expensive, and may limit generalizability. Future studies should consider hybrid designs incorporating controlled feeding during sample collection periods with ad libitum intake during washout phases.

### 6.3. Outcome Heterogeneity and the Need for Core Outcome Sets

The extraordinary diversity of reported outcome measures in exercise–microbiome trials precludes meaningful meta-analysis and hinders cross-study comparison. Some studies report alpha diversity indices (observed species, Chao1, Shannon, Simpson) without specification of rarefaction depth or phylogenetic weighting [79]. Beta diversity is visualized through principal coordinate analysis but seldom quantified through permutational multivariate analysis of variance with effect size estimation. Taxonomic relative abundances are reported at phylum, family, genus, and occasionally species levels, with inconsistent adjustment for multiple comparisons. Functional outputs range from targeted quantification of three SCFAs to untargeted metabolomics covering hundreds of metabolites [80]. The development and adoption of a core outcome set for exercise–microbiome trials, developed through consensus among international experts, is urgently required. This core set should specify minimum standards for sample collection, sequencing depth, diversity reporting, taxonomic resolution, and functional annotation.

### 6.4. Intervention Duration and Temporal Dynamics

The optimal duration of exercise interventions for inducing detectable microbial shifts remains unknown. Most published trials have employed 6- to 8-week protocols, durations selected for participant compliance and feasibility rather than microbiological rationale. Allen and colleagues demonstrated that 6 weeks is sufficient to alter butyrate production in lean individuals [8], but it remains unclear whether longer interventions would produce larger effects or whether certain slowly adapting taxa require extended exposure. Conversely, the rapid reversal of microbial changes upon detraining (within 6 weeks) suggests that the microbial ecosystem is highly responsive to the current exercise environment and that extended interventions may not confer durable advantages beyond the immediate training period.

The null finding by Davies and colleagues with a 3-week intervention raises important questions about the minimum duration required for detectable microbial changes [70]. Despite substantial metabolic improvements, no microbiome alterations were observed, suggesting either that 3 weeks is insufficient for microbial adaptation or that energy restriction and exercise in combination produce different effects than exercise alone. Time-series studies with frequent longitudinal sampling are needed to characterize the temporal dynamics of exercise-induced microbial adaptation and to identify critical windows for intervention. The 12-week interventions reported by Dupuit [71] and Torres-Peña [72] demonstrate that longer durations can yield detectable changes, though effect sizes remain modest.

### 6.5. Beyond 16S rRNA: The Imperative for Shotgun Metagenomics

The vast majority of exercise–microbiome studies to date have employed amplicon sequencing of the 16S rRNA gene, a cost-effective approach that provides taxonomic assignment to genus level with reasonable accuracy [81]. However, 16S sequencing suffers from fundamental limitations that constrain mechanistic inference. First, phylogenetic relatedness does not guarantee functional equivalence; congeneric species may possess divergent metabolic capacities and host interaction profiles [82]. Second, 16S sequencing cannot distinguish between closely related species or resolve strain-level variation, which may be critical for understanding host–microbe specificity. Third, functional inference from 16S data (e.g., PICRUSt2) provides only indirect predictions of metagenomic content and performs poorly for metabolic pathways unique to specific strains [83].

The study by Davies and colleagues exemplifies the power of shotgun metagenomics, providing strain-resolved taxonomic profiles and direct assessment of gene content [70]. Their negative findings carry greater weight because they are based on comprehensive metagenomic analysis rather than lower-resolution 16S sequencing. Similarly, the Torres-Peña study’s ability to detect species-level changes (e.g., *Escherichia fergusonii* ATCC35466) required the resolution that only metagenomics or targeted qPCR can provide [72]. The increasing accessibility and decreasing cost of shotgun metagenomics render its adoption in exercise research both feasible and imperative.

### 6.6. Inter-Individual Variability and Personalized Approaches

The observation that only 50% of participants respond to standardized exercise interventions [13], while disconcerting from a traditional group-analysis perspective, represents an opportunity for precision exercise medicine. The sources of this variability are likely multifactorial, encompassing baseline microbial composition (enterotype), host genetics (polymorphisms in SCFA receptors FFAR2/FFAR3, bile acid receptors FXR/TGR5, and pattern recognition receptors TLR4/NOD2), habitual diet, medication use, and psychosocial factors [84].

Recent studies have substantially strengthened the evidence for baseline microbiome composition as a predictor of training response. Dupuit and colleagues (2022) explicitly demonstrated that baseline microbiota composition predicted the magnitude of HIIT + RT-induced body composition changes in postmenopausal women [71]. This finding aligns with the emerging framework of exercise microbiomics, analogous to pharmacomicrobiomics, which seeks to explain variability in training responses and identify individuals most likely to benefit from specific exercise modalities. The personalized trial by AL-Elaimat and colleagues represents an important proof of concept [74]; larger trials incorporating multi-omics profiling and machine learning algorithms are now required.

The review by Liu and colleague’s frames this within 3P medicine, emphasizing the diagnostic and therapeutic potential of personalized probiotic strategies guided by quantitative assessment of microbiota and host responses [75], a framework originally articulated by Golubnitschaja and colleagues [85]. As regulatory pathways for engineered probiotics become established [76], the integration of microbiome profiling with targeted interventions becomes increasingly feasible.

### 6.7. The Gut–Muscle Axis: From Animal Models to Human Translation

The concept of a bidirectional gut–muscle axis has gained substantial traction, supported by compelling animal evidence and plausible mechanistic pathways [86]. Germ-free mice exhibit reduced skeletal muscle mass, impaired oxidative fiber typing, and diminished exercise capacity compared to conventionally colonized controls [87]. Butyrate supplementation increases muscle mass and mitochondrial biogenesis in aged mice and enhances running performance in young mice. Secondary bile acids activate TGR5 receptors on skeletal muscle, increasing energy expenditure and oxidative metabolism. Fecal microbiota transplantation transfers exercise-responsive phenotypes to sedentary recipients. Collectively, these observations establish that the gut microbiota is not merely responsive to exercise but actively contributes to exercise adaptation.

A comprehensive review by He and colleagues synthesizes the molecular mechanisms of the gut–muscle axis, identifying SCFAs, particularly butyrate, as key effectors activating AMPK-PGC-1α signaling in skeletal muscle [63]. The review notes that SCFAs contribute 12–15% of skeletal muscle total ATP production and that butyrate can enhance muscle endurance by 23% in animal models. Clinical data demonstrate that fecal butyryl-CoA transferase gene copy number correlates significantly with muscle strength in humans (*p* = 0.003).

Human evidence for the gut–muscle axis remains correlational. Cross-sectional studies have associated higher microbial diversity and butyrate-producer abundance with greater muscle mass and physical function in older adults [88]. Longitudinal exercise interventions have demonstrated concurrent improvements in muscle strength and shifts in microbial composition, but causality cannot be inferred from such designs. The Ramos et al. (2025) study in older adults provides important correlational support, demonstrating positive associations between physical activity levels, microbial diversity, and butyrate-producer abundance in the target population most relevant to sarcopenia [73]. A registered clinical trial protocol describes a planned 12-week randomised controlled trial combining Baduanjin (a traditional Chinese mind-body exercise) with resistance training in older adults with sarcopenia. This study will examine the intervention’s effects on gut microbiota composition, muscle mass, strength, and physical performance, assess short-chain fatty acid profiles, and include a parallel animal experiment using fecal microbiota transplantation in sarcopenic mice [89]. The trial aims to provide human validation for the gut-muscle axis and support the future development of microbiota-targeted exercise interventions in geriatric medicine [89].

## 7. Clinical and Translational Implications

### 7.1. Established Applications (Supported by Moderate- to High-Quality Evidence)

Irritable bowel syndrome (IBS), constipation predominant: Regular aerobic exercise (e.g., walking 30 min/day) accelerates colonic transit and reduces symptom severity. A randomized controlled trial demonstrated that physical activity improves global IBS symptoms compared to usual care [90].

Upper respiratory tract infections (URTI) in athletes: Specific probiotic strains (*Lactobacillus casei Shirota*, *Lactobacillus rhamnosus GG*, *Bifidobacterium animalis* subsp. *lactis* BL-99) reduce the incidence, duration, and severity of URTI in endurance athletes [69,91]. This aligns with the established definition and health claims for probiotics [92]. Comprehensive reviews support these benefits for athletes, though performance effects remain unproven [93]. However, a systematic review concluded that probiotics do not enhance athletic performance [94].

### 7.2. Speculative/Emerging Directions (Preclinical or Preliminary Human Evidence)

Personalized exercise prescription guided by microbiome profiling: The pilot trial by AL-Elaimat et al. (n = 10) suggests feasibility but requires replication in larger cohorts [74].

Engineered next-generation probiotics: Regulatory pathways are evolving, but no engineered probiotic has yet been approved for performance or metabolic indications [76].

Gut-muscle axis for sarcopenia: Animal models are promising, but human evidence is currently lacking. A registered clinical trial protocol by Ren et al. describes a planned 12-week intervention combining Baduanjin with resistance training in older adults with sarcopenia [89]. As no results are yet available, this axis remains investigational and should not be used to guide clinical therapy.

Metabolic dysfunction-associated steatotic liver disease (MASLD): Exercise reduces hepatic steatosis independent of weight loss, but a direct microbiota-mediated mechanism in humans is not proven [95]. Similarly, exercise may benefit patients with quiescent inflammatory bowel disease, though evidence remains preliminary [96].

### 7.3. Cautionary Note

Many of the above applications are supported by preliminary evidence only. Clinicians and practitioners should not implement microbiome-guided exercise prescription or probiotics for performance enhancement outside of research settings until adequately powered, preregistered confirmatory trials are published.

## 8. Limitations of This Review

This narrative review, while comprehensive, is subject to several limitations. First, the search strategy, although systematic in approach, was not registered in PROSPERO and did not follow PRISMA guidelines for systematic reviews. Quantitative synthesis (meta-analysis) was not performed due to substantial heterogeneity in study design, participant characteristics, exercise interventions, and outcome measures. Second, the review is limited to English- and Persian-language publications, potentially excluding relevant studies in other languages. Third, the rapid pace of publication in this field means that studies published after February 2025 are not included. Fourth, the review emphasizes mechanistic and translational aspects; readers seeking detailed methodological guidance on 16S rRNA sequencing, metagenomics, or metabolomics are referred to specialized resources [81,97]. Finally, the authors’ own research interests may introduce unconscious bias in the selection and interpretation of evidence.

## 9. Conclusions

The past decade has witnessed extraordinary progress in understanding the bidirectional interactions between exercise and the gut microbiome. The recent period has been particularly transformative, characterized by methodologically rigorous studies that have both confirmed and challenged prevailing assumptions. It is now established that:

Exercise can modify gut microbiota composition and function independently of diet. This conclusion is definitively supported by animal studies with controlled feeding and fecal microbiota transplantation and is strongly suggested by human trials with dietary control. However, human effect sizes are modest and highly variable; the independent effect in free-living humans is still debated.

Exercise effects are intensity-dependent, transient, and reversible. Moderate-intensity continuous training reliably increases microbial diversity, enriches health-associated taxa (*Akkermansia*, *Faecalibacterium*, *Roseburia*), and enhances intestinal barrier function. High-intensity interval training acutely perturbs the ecosystem but, following adaptation, stimulates butyrate production through lactate cross-feeding. Recent evidence suggests that HIIT may also increase potentially pathobiont taxa (e.g., *E. fergusonii*), indicating that intensity-dependent effects are complex and require individualized risk–benefit assessment [72]. Detraining reverses microbial gains within 6 weeks.

The gut microbiota contributes to exercise adaptation in animal models. Germ-free status impairs exercise capacity, antibiotic treatment abolishes training adaptations, FMT from trained donors confers barrier protection to sedentary recipients [61], and butyrate supplementation enhances performance. Human translation of the gut–muscle axis remains incomplete.

Inter-individual variability is substantial and non-random. Approximately 50% of participants exhibit minimal microbial responses to standardized exercise protocols. Baseline microbiota composition predicts training response, as demonstrated in postmenopausal women [71] and metabolic syndrome patients [74]. This variability creates opportunities for personalized exercise prescription but also requires larger sample sizes in future trials.

Critical knowledge gaps remain. Definitive evidence linking exercise-induced microbial shifts to improved human athletic performance is lacking. The null finding by Davies and colleagues demonstrates that substantial metabolic improvements can occur without detectable microbiome changes [70], emphasizing that the microbiome is one component of a multi-system response rather than a necessary mediator. The optimal exercise dose (frequency, intensity, time, type) for microbiome benefits is undefined. Mechanisms established in animals require human validation. The therapeutic potential of the gut–muscle axis in sarcopenia, cachexia, and age-related frailty awaits confirmation in adequately powered randomized controlled trials. 

The field now stands at an inflection point. The era of descriptive, underpowered, 16S-based observational studies must yield to hypothesis-driven, adequately powered, multi-omics randomized controlled trials with mechanistic validation. Simultaneously, the immense inter-individual variability in responses must be embraced as a gateway to personalized exercise medicine rather than dismissed as statistical noise. The emergence of 3P medicine frameworks [75,85] and next-generation probiotic strategies [76] provides a roadmap for translation. If these methodological and conceptual transitions are successfully navigated, the next quinquennium will witness the translation of the exercise–gut microbiome axis from academic curiosity to clinical reality—improving health, performance, and well-being across the lifespan.

## Figures and Tables

**Table 1:** Summary of Animal Studies (Murine Models) on Exercise and Gut Microbiota.

Reference (Country, Year)	Study Design	Sample Size	Participant Characteristics	Exercise Intervention	Dietary Control	Quality Score (SYRCLE)	Key Findings Related to Gut Microbiota
**Matsumoto et al. (Japan, 2008) [57]**	Experimental	20	Male Wistar rats	Voluntary wheel running, 5 weeks	Standard chow	Moderate	↑ Cecal butyrate proportion, ↑ *Bacteroidota*, ↓ *Bacillota*; first demonstration of exercise-induced butyrate increase
**Evans et al. (USA, 2014) [58]**	Experimental	24	Male C57BL/6 mice, high-fat diet	Treadmill running, 12 weeks	Standard chow (pair-fed)	Moderate	↑ Alpha diversity, ↑ *Bacteroidota*/*Bacillota* ratio, ↓ intestinal inflammation; exercise partially overrides diet effects
**Denou et al. (Canada, 2016) [59]**	Experimental + FMT	30	Male C57BL/6 mice	Voluntary wheel running, 6 weeks; FMT to germ-free recipients	Standard chow	Low	Altered microbiota composition; FMT from active donors → ↑ glucose tolerance in recipients; causal evidence
**Lamoureux et al. (Canada, 2017) [64]**	Experimental	20	Male Wistar rats	High-intensity interval training, 6 weeks	Standard chow	Moderate	↑ Butyrate-producing bacteria, ↑ intestinal barrier integrity, ↓ plasma LPS; HIIT-specific effects
**Campbell et al. (USA, 2019) [60]**	Experimental	40	Female C57BL/6 mice, lean vs. obese	Voluntary wheel running, 6 weeks	Standard chow	Moderate	Strain- and obesity-dependent effects; microbial changes attenuated in obese mice
**Huang et al. (China, 2017) [62]**	Experimental	Not specified	Pregnant rats, offspring	Maternal dietary butyrate supplementation (1%)	Standard chow	Low	↑ ATP content, ↑ mitochondrial DNA copy number, ↑ PGC-1α expression in offspring muscle; demonstrates butyrate’s role in programming mitochondrial biogenesis via GPR43/41
**Liu et al. (China, 2025) [61]**	Experimental + FMT	40	Male C57BL/6 mice	14 weeks moderate training; FMT to sedentary recipients	Standard chow	Low	FMT from trained donors ↑ ZO-1 (*p* < 0.05), ↓ LPS (*p* < 0.01), ↓ IL-6 (*p* < 0.01), ↑ sIgA (*p* < 0.01) in recipients after acute exhaustive exercise; demonstrates transferable barrier protection
**Kalantari et al. (Iran, 2025) [50]**	Experimental	32	Aged male Wistar mice with DSS-induced colitis	8 weeks HIIT (85–100% VO_2_max) vs. MICT (70–75% VO_2_max)	Standard chow	Moderate	HIIT ↑ Shannon index (3.2 ± 0.2 vs. 2.5 ± 0.3), ↑ *Lactobacillus* and *Bifidobacterium*, ↓ *E. coli* (*p* < 0.001); HIIT effects significantly greater than MICT (*p* = 0.04)

FMT, fecal microbiota transplantation; HIIT, high-intensity interval training; LPS, lipopolysaccharide; MICT, moderate-intensity continuous training; sIgA, secretory immunoglobulin A; ↑ increase; ↓ decrease.

**Table 2:** Summary of Human Studies on Exercise and Gut Microbiota.

Reference (Country, Year)	Study Design	Sample Size	Participant Characteristics	Exercise Intervention	Dietary Control	Sequencing Method	Key Findings Related to Gut Microbiota
**Clarke et al. (Ireland, 2014) [65]**	Cross-sectional	40	Elite rugby players vs. sedentary controls	—	Not controlled	16S rRNA	↑ Alpha diversity, ↑ 40 bacterial taxa, ↑ *Akkermansia*, ↑ butyrate producers; first human evidence
**Bressa et al. (Spain, 2017) [36]**	Cross-sectional	48	Active vs. sedentary women	—	Not controlled	16S rRNA	↑ *Faecalibacterium prausnitzii*, ↑ *Roseburia hominis*, ↑ *Akkermansia muciniphila*; female-specific data
**Estaki et al. (Canada, 2016) [19]**	Cross-sectional	39	Healthy adults	—	Not controlled	16S rRNA	VO_2_max positively correlated with alpha diversity and butyrate-producer abundance; dose–response evidence
**Barton et al. (Ireland, 2018) [7]**	Cross-sectional (metagenomics)	40	Rugby players vs. controls	—	Not controlled	Shotgun	↑ Amino acid biosynthesis, ↑ carbohydrate metabolism, ↑ SCFA potential; strain-level resolution
**Allen et al. (USA, 2018) [8]**	RCT, controlled diet	32	Sedentary lean/obese adults	6 wk endurance, 60–75% HRR	Controlled feeding	16S rRNA	↑ *Faecalibacterium*, ↑ butyrate (lean only); reversal with detraining; BMI-dependent effects
**Munukka et al. (Finland, 2018) [13]**	RCT	24	Sedentary overweight women	6 wk light-moderate resistance	Ad libitum (habitual)	Shotgun	↑ *Akkermansia*, ↓ *Proteobacteria*; only 50% responded; inter-individual variability
**Cronin et al. (Ireland, 2019) [67]**	RCT	90	Overweight/obese adults	8 wk endurance ± whey protein	Ad libitum (habitual)	16S rRNA + shotgun	No major taxonomic shifts; trend toward ↑ diversity; dietary confounding
**Davies et al. (UK, 2025) [70]**	RCT, controlled diet	30	Overweight/obese sedentary adults	3 wk energy restriction + vigorous walking (70% VO_2_peak)	Controlled feeding	Shotgun	Significant metabolic improvements but NO changes in α/β diversity or taxonomy; demonstrates microbiome-independent pathways
**Dupuit et al. (France, 2022) [71]**	RCT	17	Postmenopausal overweight/obese women	12 wk HIIT + RT	Ad libitum (habitual)	16S rRNA	No Δα-diversity or taxonomy; ↓ visceral fat, ↑ fitness; baseline microbiota predicted individual responses
**Torres-Peña et al. (Spain, 2023) [72]**	RCT	33	T2D, overweight/obese adults	12 wk HIIT vs. MICT vs. control	Ad libitum (habitual)	16S rRNA	Both HIIT/MICT ↑ *Marseillibacter massiliensis* (r = 0.58 with richness); HIIT also ↑ *E. fergusonii* (potential risk)
**Ramos et al. (UK, 2025) [73]**	Cross-sectional	60	Older adults (65–85 y)	Accelerometer-measured PA	Not controlled	16S rRNA	MVPA positively associated with α-diversity and butyrate-producer abundance; extends dose–response to geriatric population
**AL-Elaimat et al. (Jordan, 2025) [74]**	RCT (personalized)	10	Metabolic syndrome patients	12 wk personalized vs. standard exercise	Ad libitum (habitual)	16S rRNA	Personalized group ↑ microbial richness, ↑ butyrate producers, ↑ metabolic improvements; proof-of-concept for microbiome-guided prescription (preliminary)

RCT, randomized controlled trial; HRR, heart rate reserve; HIIT, high-intensity interval training; RT, resistance training; MICT, moderate-intensity continuous training; T2D, type 2 diabetes; MVPA, moderate-to-vigorous physical activity; SCFA, short-chain fatty acid; ↑ increase; ↓ decrease.

## Data Availability

The original contributions presented in this study are included in the article. Further inquiries can be directed to the corresponding author.

## Supplementary Materials

The following supporting information can be downloaded at: https://www.mdpi.com/article/10.3390/nu18101565/s1, Table S1. Methodological quality assessment of included studies.
