# Supplementary material for: Exercise and the Gut Microbiome: From Mechanisms to Clinical Applications
Source: Nutrients. 2026 May 14;18(10):1565. doi: 10.3390/nu18101565 (PMC13209957; doi:10.3390/nu18101565)
Supplement: Supplementary file 1 [file nutrients-18-01565-s001.zip › nutrients-4200259-supplementary.pdf]

Supplementary Table S1. Methodological quality assessment of included studies.

Study (First Author, Year)	Study Design	Quality Assessment Tool	Overall Risk of Bias / Quality Rating	Key Limitations Noted
**Animal studies**				
**Matsumoto et al. 2008 [57]**	Experimental (rat)	SYRCLE's RoB	Moderate	Randomisation not described; blinding not reported
**Evans et al. 2014 [58]**	Experimental (mouse)	SYRCLE's RoB	Moderate	Allocation concealment unclear
**Denou et al. 2016 [59]**	Experimental + FMT (mouse)	SYRCLE's RoB	Low	Clear randomisation; FMT controlled; outcome assessment blinded
**Lamoureux et al. 2017 [64]**	Experimental (rat)	SYRCLE's RoB	Moderate	Sample size small (n=10/group); no blinding
**Campbell et al. 2019 [60]**	Experimental (mouse)	SYRCLE's RoB	Moderate	No blinding; obesity model well described
**Huang et al. 2017 [62]**	Experimental (rat, maternal)	SYRCLE's RoB	Low	Clear intervention; offspring outcomes objective
**Liu et al. 2025 [61]**	Experimental + FMT (mouse)	SYRCLE's RoB	Low	FMT protocol rigorous; outcome assessors blinded
**Kalantari et al. 2025 [50]**	Experimental (mouse, DSS colitis)	SYRCLE's RoB	Moderate	Randomisation stated but allocation not detailed
**Human studies**				
**Clarke et al. 2014 [65]**	Cross-sectional	N/A (observational)	Moderate (non-comparative)	No dietary control; residual confounding
**Bressa et al. 2017 [36]**	Cross-sectional	N/A	Moderate	Self-reported physical activity; no diet control
**Estaki et al. 2016 [19]**	Cross-sectional	N/A	Moderate	VO_2max measured, but diet not controlled
**Barton et al. 2018 [7]**	Cross-sectional (metagenomics)	N/A	Moderate	Elite athletes vs. controls; dietary differences
**Allen et al. 2018 [8]**	RCT, controlled diet	Cochrane RoB 2	**Low**	Controlled feeding; randomisation; blinding of outcome assessment
**Munukka et al. 2018 [13]**	RCT	Cochrane RoB 2	**Some concerns**	No dietary control; 50% non-responders
**Cronin et al. 2019 [67]**	RCT	Cochrane RoB 2	**Some concerns**	No dietary control; wide BMI range
**Davies et al. 2025 [70]**	RCT, controlled diet	Cochrane RoB 2	**Low**	Controlled feeding; shotgun metagenomics; outcome assessors blinded
**Dupuit et al. 2022 [71]**	RCT	Cochrane RoB 2	**Some concerns**	No dietary control; small sample (n=17)

**Torres-Peña et al. 2023 [72]**	RCT (conference abstract)	Cochrane RoB 2	**High** (preliminary)	Abstract only; small sample (n=33); no dietary control
**Ramos et al. 2025 [73]**	Cross-sectional	N/A	Moderate	Accelerometry objective; no diet control
**AL-Elaimat et al. 2025 [74]**	RCT (personalised)	Cochrane RoB 2	**High**	Very small sample (n=10); no blinding; preliminary

Notes: SYRCLE's RoB = SYRCLE's Risk of Bias tool for animal studies (low, moderate, high). Cochrane RoB 2 = Cochrane Risk of Bias 2 tool for randomised controlled trials (low, some concerns, high). Cross-sectional studies were not formally assessed with a risk of bias tool but are noted for confounding. FMT = faecal microbiota transplantation.
